# Evaluation of Concrete Compressive Strength Prediction Using the Maturity Method Incorporating Various Curing Temperatures and Binder Compositions

**DOI:** 10.3390/ma17235794

**Published:** 2024-11-26

**Authors:** Gum-Sung Ryu, Gi-Hong An, Yong-Sik Yoon, Ji-Young Kim, Sung Choi

**Affiliations:** 1 Department of Structural Engineering Research, Korea Institute of Civil Engineering and Building Technology, Goyang 10223, Republic of Korea; ryu0505@kict.re.kr (G.-S.R.); agh0530@kict.re.kr (G.-H.A.); 2 Korea Peninsula Infrastructure Special Committee, Korea Institute of Civil Engineering and Building Technology, Goyang 10223, Republic of Korea; humbleyys@kict.re.kr; 3 Smart Hi-Tech Group, SAMSUNG E&A, 26, Sangil-ro 6 gil, Gangdong-gu, Seoul 05288, Republic of Korea; jy0421.kim@samsung.com; 4 Department of Civil Engineering, Kyungdong University, 27, Gyeongdongdaehak-ro, Yangju-si 11458, Republic of Korea

**Keywords:** compressive strength, maturity method, curing temperature, binder content, predictive modeling

## Abstract

This study aims to systematically analyze the effects of different curing temperatures, unit binder content, and the mixture ratios of ground granulated blast-furnace slag and fly ash based on ordinary Portland cement in binders on the development of concrete compressive strength. Particularly, the study evaluates strength characteristics by calculating the maturity equivalent to 28 days of curing at 20 °C. A model based on the relationship between maturity and strength was applied to predict the compressive strength, and the experimental data were analyzed to derive strength coefficients for each variable. The results showed that at a low temperature of 5 °C, the actual strength was lower than the predicted strength, leading to higher error rates. In contrast, at temperatures of 20 °C and 40 °C, the coefficient of determination (R^2^ > 0.90) for the predictive equation was high, and the error rates were reduced to within 10%. The study demonstrates that by combining the maturity method with the strength–maturity relationship, the concrete compressive strength can be effectively predicted under specific curing and binder design conditions.

## 1. Introduction

Predicting the compressive strength of concrete structures remains a significant challenge due to the complexity of various environmental factors and binder compositions. Test specimens produced under controlled laboratory conditions often fail to reflect the actual strength of structures accurately, as real concrete structures in the field are exposed to a wide range of curing conditions. In particular, the temperature distribution within a structure varies due to the different heat of hydration depending on its shape and location, directly impacting the strength development [1]. As a result, even with the same materials, the strength can vary based on location and condition, making it difficult to predict the actual strength of structures solely based on simple experimental results.

One promising approach to address this issue is the maturity method, which is gaining attention for strength prediction. The maturity method predicts the strength based on the heat of hydration generated during curing, offering a more accurate reflection of the curing environment in actual structures. Early research introduced various models to explain the relationship between maturity and strength [2,3,4]. The Nurse–Saul model assumed a linear relationship between maturity and strength, allowing relatively simple calculations. However, it exhibited significant errors under low-temperature curing conditions [5,6]. Subsequently, the Arrhenius model enhanced the accuracy of prediction by considering the nonlinear effects of temperature; however, its complex calculations limited its practical application in the field [7,8]. These strength prediction model enabled more precise predictions but showed limitations in environments with rapid temperature changes. The logarithmic function model, introduced recently, has the advantage of explaining the nonlinear relationship between the maturity index and strength and enabling accurate predictions even with limited data. These models have contributed to reducing experimental costs and time by predicting strengths focused on a wide range of concrete mixtures and conditions [9,10,11].

Recent studies have focused on improving the accuracy of concrete strength prediction using the maturity method. For instance, Kazemifard et al. predicted the strength of self-compacting concrete using the maturity method, achieving over 94% accuracy while maintaining reliable results without the need for additional adjustments [12]. Cheng et al. investigated the effect of the water-to-binder ratio (W/B) on concrete compressive strength, and predicted the strength by effectively reflecting the changes in these variables using the maturity method. The study revealed that the strength increased as the W/B decreased, indicating the high sensitivity of the maturity method to these conditions [13]. Kampli et al. introduced an IoT-based real-time monitoring system to predict in situ concrete strength using the maturity method, showing a high correlation between the predicted and actual strengths [14]. Additionally, Miller et al. conducted a study on real-time monitoring of early-age concrete compressive strength utilizing an IoT-based system. This study proposed a system that effectively predicts the early-age compressive strength of concrete through the automation of the maturity method, demonstrating stable performance under various site conditions [15]. These studies suggest that the maturity method can be used as a highly valuable tool for mix design and strength prediction, and show that an IoT-based monitoring system can further improve the accuracy of these predictions. In addition, from an environmental perspective, the maturity method also plays a significant role. For instance, Imran et al. developed a multivariate polynomial regression model incorporating the maturity method to predict the compressive strength of eco-friendly concrete mixtures, demonstrating higher accuracy compared to traditional prediction models [16].

These studies illustrate the development of the maturity method and its applicability, contributing to improving prediction accuracy across diverse conditions and concrete mixtures. However, several challenges still remain when applying these methods to real structures. In practice, it is often difficult to maintain the homogeneity of field concrete, and varying environmental factors and curing conditions can cause discrepancies between theoretical predictions and actual data. Additionally, errors can occur if the time–temperature records used in calculating the maturity index are not accurately managed. These issues suggest the need for further research and improvements to ensure reliable predictions when applying the maturity method to real structures.

This study aims to address the limitations of the existing maturity method by proposing a novel strength prediction model that comprehensively considers various curing temperatures (5 °C, 20 °C, 40 °C) and binder compositions. In particular, the relationship between maturity and compressive strength was quantitatively analyzed using a range of binders, including ground granulated blast-furnace slag (GGBFS) and fly ash (FA). This approach enabled the model to account for the effects of temperature and binder composition on strength development, improving its structural accuracy and predictive capabilities. Furthermore, the study systematically derived strength coefficients for each mix condition by establishing mathematical relationships between the maturity index and compressive strength based on experimental data. These coefficients were tailored to reflect varying mix designs and curing environments, enhancing the reliability and adaptability of the model. Rather than limiting the analysis to laboratory conditions, the research incorporated diverse mix proportions designed to simulate real-world field conditions, thereby broadening the scope of the model’s application. To validate the effectiveness of the proposed model, mock-up specimens (slabs and beams) were prepared under conditions similar to those of actual structures. These specimens were used to evaluate how accurately the model could predict strength development in practical settings. By comparing the predicted strength values with the actual measured strengths, the accuracy and applicability of the model were thoroughly assessed, demonstrating its potential for real-world implementation.

## 2. Experimental Program

### 2.1. Raw Materials

The following is an explanation of Table 1, which lists the physical properties and chemical compositions of cement, GGBFS, and FA. All the cement, GGBFS, and FA used in this study comply with Korean Standards (KS). The cement is a Type 1 ordinary Portland cement (OPC, SAMPYO, Samcheok, Gangwon-do, Republic of Korea) in accordance with KS F 5201. The GGBFS was supplied by SAMPYO (Pyeongtaek, Gyeonggi-do, Republic of Korea) and conforms to the Type 3 specification in KS F 2563, while the FA, provided by SAMPYO (Boryeong, Chungcheongnam-do, Republic of Korea), meets the Type 2 specification in KS L 5405 [17,18,19]. Table 1 provides detailed information on the density, specific surface area, and major chemical components of each material. First, looking at the physical properties, cement has the highest density at 3.152 g/cm^3^, followed by GGBFS at 2.908 g/cm^3^, and FA at 2.203 g/cm^3^. In terms of specific surface area, GGBFS has the highest value at 468 m^2^/kg, followed by cement at 331 m^2^/kg, and FA at 322 m^2^/kg. The chemical composition analysis shows that cement contains SiO_2_ (12.8%), Al_2_O_3_ (3.5%), Fe_2_O_3_ (5.2%), and CaO (69.6%), and the high CaO content contributes to the strong binding properties of cement. GGBFS contains SiO_2_ (34.6%), Al_2_O_3_ (14.3%), Fe_2_O_3_ (0.6%), and CaO (43.4%), especially high SiO_2_ and Al_2_O_3_, indicating that GGBFS provides additional reactivity when mixed with cement. FA contains SiO_2_ (56.8%), Al_2_O_3_ (22.8%), Fe_2_O_3_ (6.9%), and CaO (3.5%), with avery high SiO_2_ and Al_2_O_3_ content. This suggests that FA possesses strong pozzolanic properties, enhancing strength through its reaction with cement.

**Figure 1:**
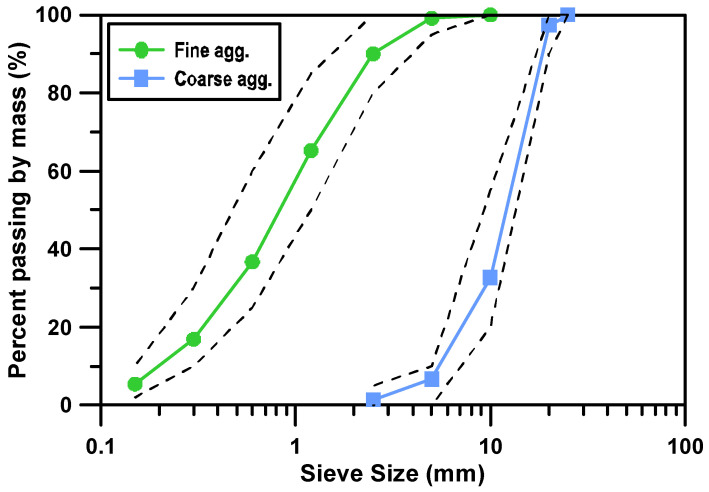
Particle size distribution of fine and coarse aggregates.

Figure 1 illustrates the particle size distribution of fine and coarse aggregates, showing how well each aggregate meets the KS [20]. The dashed lines within the graphs indicate the upper and lower bounds of the aggregate gradation as stipulated by the KS, and the green and blue solid lines represent the actual particle size distributions of the fine and coarse aggregates used in the experiment, respectively.

In the particle size distribution graph for the fine aggregates, the green solid line is located between the upper and lower dashed lines of the KS. This indicates that the fine aggregates used in the experiment have a particle size distribution that meets the KS. In other words, the fine aggregates are uniformly distributed, providing the appropriate conditions for use in concrete mixtures. Similarly, in the particle size distribution graph for the coarse aggregates, the blue solid line is also located between the upper and lower dashed lines of the KS. This demonstrates that the coarse aggregates used in the experiment also have a particle size distribution that meets the KS. The uniform distribution of coarse aggregates is suitable for ensuring the strength and durability of concrete.

### 2.2. Mix Proportion

The concrete mix proportions used in this study are shown in Table 2. The concrete mixtures are classified as B33, B37, and B41 according to the binder content, which corresponds to a unit binder weight of 330 kg/m^3^, 370 kg/m^3^, and 410 kg/m^3^, respectively. Based on these mixtures, additional mix designs incorporating various supplementary cementitious materials (SCMs) were conducted. B41_S25 and B41_S50 represent mixtures in which 25% and 50% of the 410 kg/m^3^ binder weight were replaced with GGBFS, while B41_F15 and B41_F30 represent mixtures in which 15% and 30% of the 410 kg/m^3^ binder weight were replaced with FA, respectively.

For all mixtures, the water-to-binder ratio (W/B) and sand-to-aggregate ratio (S/a) were adjusted according to the changes in binder content. A high-range water-reducing agent with air-entraining properties was used to ensure that the slump was maintained within 180 mm and the air content was kept at 4.5%, in accordance with the KS F 4009 [21]. These mix conditions were optimized to maintain consistency across the experiments and achieve the desired performance. The mix proportions in Table 2 shows the unit weights of water, binder (cement, GGBFS, and FA), fine aggregate, and coarse aggregate for each mixture, which were used to evaluate the performance of the concrete and predict compressive strength using the maturity method. Based on these mix designs, this study aimed to analyze the relationship between concrete maturity and compressive strength under various conditions.

### 2.3. Experimental Method

**Figure 2:**
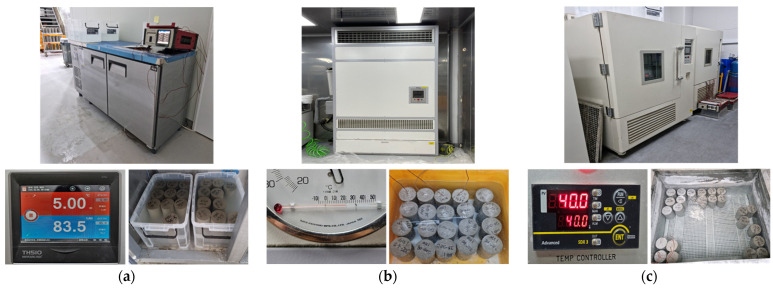
Overview of concrete curing environments and specimen curing conditions: (**a**) curing in 5 °C environment; (**b**) curing in 20 °C environment; and (**c**) curing in 40 °C environment.

Figure 2 illustrates the equipment and procedures used to cure concrete specimens under various temperature conditions. The specimens were kept at constant temperatures and subjected to water curing to analyze their characteristics under each condition. Figure 2a shows the refrigeration unit used for curing at 5 °C. The unit was set to maintain an internal temperature of 5 °C, and the specimens were cured in molds for two days. After demolding, the specimens were water-cured in a tank maintained at 5 °C. Temperature gauges were embedded in the specimens to accurately measure the internal temperature. Figure 2b shows the temperature and humidity-controlled room used for curing at 20 °C. This room was designed to maintain a constant temperature and humidity, providing optimal curing conditions. The specimens were cured in molds for one day, then demolded and water-cured in a tank maintained at 20 °C. At this time, temperature gauges were embedded in the specimens to monitor precise temperature changes. Figure 2c shows the high-temperature chamber used for curing at 40 °C. The chamber was set to 40 °C, providing high-temperature curing conditions. The specimens were cured in molds for one day, then demolded and water-cured in a tank maintained at 40 °C. Temperature gauges were also embedded in these specimens to continuously monitor the internal temperature. The equipment and procedures used for each curing condition were designed to optimize the relationship between concrete hydration reactions and strength development.

**Figure 3:**
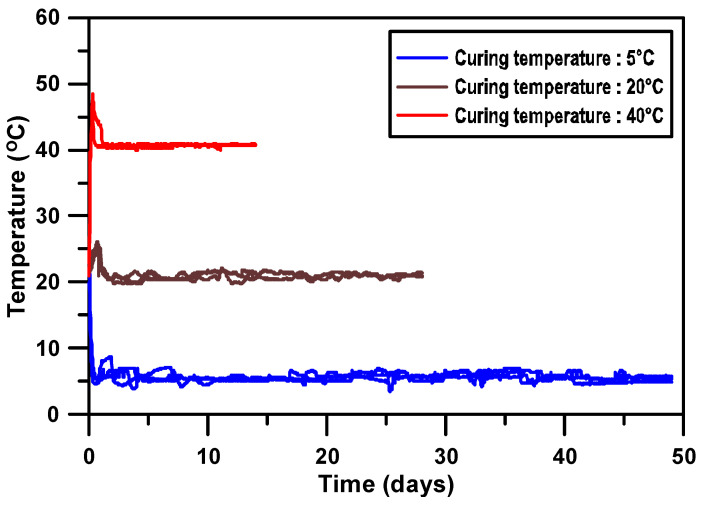
Internal temperatures of concrete specimens curing at 5 °C, 20 °C, and 40 °C.

Figure 3 illustrates the internal temperature changes of concrete specimens cured under three different temperature conditions (5 °C, 20 °C, and 40 °C). Due to the inability to install temperature gauges within the specimens used for compressive strength testing, temperature gauges were embedded in nine representative specimens specifically for measuring the concrete temperature. All specimens exhibited rapid temperature changes immediately after mixing due to the initial hydration reaction. The specimens cured at 5 °C showed a rapid decrease in temperature from the initial 23 °C to 5 °C, and then stabilized near 5 °C within one day. The specimens cured at 20 °C exhibited a gradual decline from 23 °C to 20 °C, also stabilizing within one day. The specimens cured at 40 °C experienced a sharp increase from 23 °C to 40 °C, stabilizing within one day. After the initial rapid temperature fluctuations, each specimen stabilized according to the curing temperature, allowing for the calculation of the maturity index. The maturity index, defined as the product of curing temperature and time, plays a crucial role in the hydration reaction and strength development of concrete.

The compressive strength test was conducted based on the maturity of the specimens, which were cured under different temperature conditions. The compressive strength test was performed in accordance with ASTM C109-16a [22]. The specimens used for the compressive strength test were cylindrical, with a diameter of 100 mm and a height of 200 mm. The compressive strength results were averaged from three specimens.

### 2.4. Mock-Up Test

**Figure 4:**
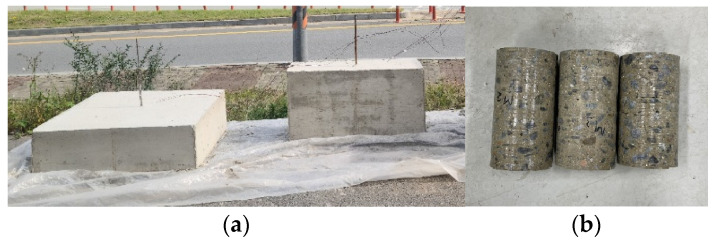
Specimens for mock-up test and core samples for testing: (**a**) slab and beam; and (**b**) coring specimens.

To verify the proposed maturity-based compressive strength prediction model, mock-up test specimens were prepared as shown in Figure 4. The mock-up specimens consisted of two types: a slab (1.0 m × 0.2 m × 1.0 m) and a beam (0.4 m × 0.4 m × 1.0 m), both designed to evaluate the applicability of the prediction model across various structural members. The concrete mix used in these specimens was consistent with the B41 mix specified in Table 2. To compare the compressive strength predicted by the maturity method with the actual compressive strength of the concrete structures, internal temperatures were measured at three points along the height of each specimen—top, middle, and bottom. After approximately 12 days, when sufficient strength was expected to have developed, core samples were extracted from each specimen. At this point, the maturity was calculated based on the temperature history of the structures, and the compressive strength of these core samples was measured and compared to the predicted values.

### 2.5. Maturity Index and Logarithmic Function Model

The maturity index is an important indicator used to predict the early strength development of concrete. The strength of concrete increases over time through a hydration process, and this process is significantly influenced by the temperature and time [23]. The maturity index integrates these time and temperature effects into a single value to evaluate the strength development of concrete. The maturity index $M_{\left(t,T\right)}$ is calculated by summing the differences between the average temperature of the concrete and a reference temperature over specific time intervals. It is generally expressed as follows:

(1) $$M_{\left(t,T\right)}=\sum \left(T_{a}-T_{0}\right)\times \Delta t$$

Here, $T_{a}$ represents the average temperature of the concrete, $T_{0}$ is the reference temperature (−10 °C), and Δt is the time interval. This index indicates how long the concrete has been cured at a specific temperature, which is crucial for predicting its strength development. Various strength prediction models have been developed through extensive research; each result explained the relationship between strength and maturity under specific conditions [24,25]. These models apply different mathematical approaches to explain the strength development of concrete. Among them, the logarithmic function model predicts the concrete strength based on the logarithmic relationship between the maturity index and strength, which is expressed by the following equation:

(2) $$S=a+b\log\left(M_{\left(t,T\right)}\right)$$

where S is the strength of the concrete, a and b are constants determined from experimental data, and $M_{\left(t,T\right)}$ is the maturity index. This model uses a logarithmic function to describe the nonlinear relationship between the maturity index and strength. The logarithmic function model is relatively simple, making it easy to calculate and apply [26]. This simplicity is advantageous for analyzing experimental data and practical application on actual construction sites. Additionally, this model is applicable to various concrete mixtures and conditions, making it particularly useful for predicting early strength. Its broad applicability to different materials and mix ratios enhances its generalizability. Thus, this model can be used for real-time strength monitoring on construction sites and structural safety evaluations, and enhances construction efficiency while ensuring structural safety.

## 3. Results and Discussion

### 3.1. Maturity and Compressive Strength Analysis Based on Curing Temperature

Table 3 and Table 4 present the compressive strength results of concrete cured at different temperatures (5 °C, 20 °C, and 40 °C) with a focus on analyzing the strength development using the maturity concept. The maturity index, which integrates the effects of time and temperature, serves as a key indicator in predicting the compressive strength of concrete. In this study, the compressive strength of concrete was measured at five maturity levels, calculated based on temperature–time relations, under each curing condition. Despite variations in curing temperature, this approach allows for comparing compressive strengths under different conditions on a common maturity scale.

Table 3 organizes the maturity ($M_{\left(t,T\right)}$) and compressive strength results by binder content. The results demonstrate that, regardless of the temperature, an increase in binder content generally leads to higher compressive strength across all maturity levels. This is particularly evident at higher maturity values, where the strength differences between mixtures become more pronounced. At 20 °C, which represents standard curing conditions, compressive strengths at maturity levels around 800 MM consistently show significant gains, confirming the influence of the binder content on strength development. Table 4 categorizes the results by binder type, specifically examining mixtures incorporating GGBFS and FA. The influence of SCMs on strength development is evident, with GGBFS-blended mixtures (B41_S25 and B41_S50) exhibiting more gradual strength development at early stages compared to plain cement mixtures. However, at higher maturity levels, GGBFS mixtures display competitive or even superior strengths, reflecting their potential for long-term strength gain. FA-blended mixtures (B41_F15 and B41_F30), on the other hand, show slower strength development overall, particularly at lower curing temperatures like 5 °C. This behavior aligns with the pozzolanic reaction of FA, which is activated at later stages, leading to strength gains primarily at higher maturities [27].

The results highlight the applicability of the maturity concept in analyzing compressive strength across diverse curing conditions and binder compositions. By normalizing the strength development through maturity, it is possible to predict the performance of concrete mixtures even under non-standard curing conditions [28]. The analysis further demonstrates that the maturity index effectively captures the influence of temperature on hydration and strength gain, allowing for a comprehensive assessment of both the short-term and long-term behavior of concrete under various environmental conditions.

### 3.2. Compressive Strength and Maturity Analysis Based on Logarithmic Regression Models

**Figure 5:**
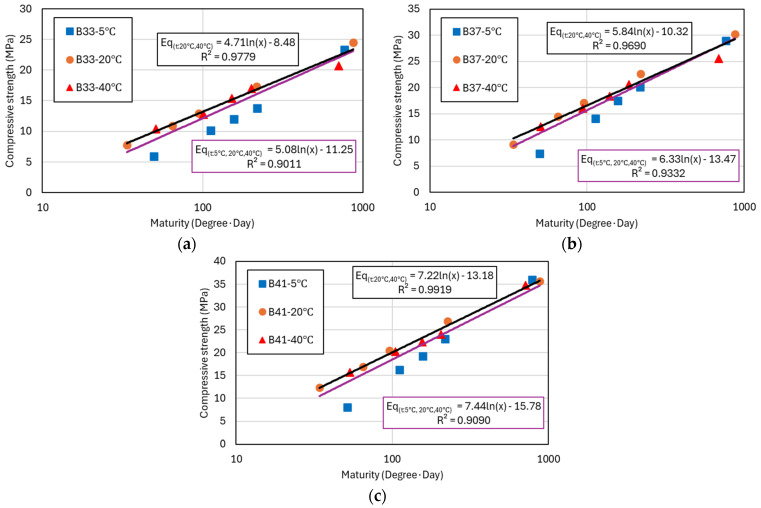
Compressive strength and maturity analysis for binder unit weight. (**a**) B33; (**b**) B37; and (**c**) B41.

**Figure 6:**
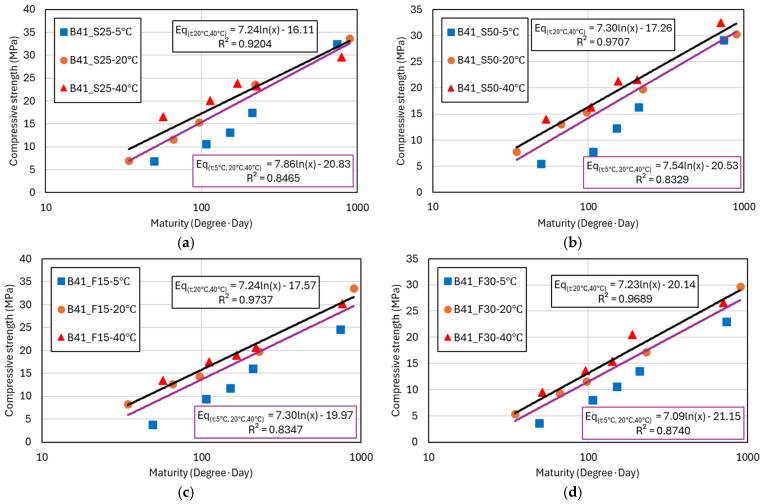
Compressive strength and maturity analysis for different W/B ratios. (**a**) B41_S25; (**b**) B41_S50; (**c**) B41_F15; and (**d**) B41_F30.

Figure 5 and Figure 6 present the regression analysis results using the logarithmic function model for the compressive strength data previously shown in Table 3 and Table 4. The graphs display compressive strength results measured at similar maturity levels for concrete cured at 5 °C, 20 °C, and 40 °C. Each graph includes two regression trendlines: a purple line representing the regression analysis that includes compressive strength data from all curing temperatures (5 °C, 20 °C, 40 °C), and a black line representing the regression excluding the compressive strength data from 5 °C curing, focusing only on the 20 °C and 40 °C data. From the graphs, it is evident that the compressive strength of concrete cured at 5 °C is significantly lower compared to that of concrete cured at 20 °C and 40 °C, even at similar maturity levels. This discrepancy highlights the negative impact of low temperatures on strength development, likely due to slower hydration processes [29]. To account for this, the black trendline was introduced, analyzing only the data from 20 °C and 40 °C curing conditions.

The analysis results show that the black trendline exhibits a high coefficient of determination ($R^{2}$) of over 90%, indicating a strong fit for the compressive strength results at 20 °C and 40 °C. In contrast, the purple trendline, which includes data from all temperatures, shows $R^{2}$ values ranging from 83% to 93%, suggesting greater variance and a larger margin of error in predicting compressive strength using the logarithmic model when low-temperature data are included [30]. This phenomenon is more pronounced in mixtures containing GGBFS and FA, as shown in Figure 6. The incorporation of SCMs tends to delay strength development, particularly at lower temperatures, further amplifying the observed discrepancies in the regression analysis [27,30]. The logarithmic function model is represented as Equation (2), where the constants a and b vary depending on the binder content and type. Through this, a compressive strength prediction model based on the maturity concept can be developed. Maturity is a key concept that quantitatively assesses the interaction between temperature and time, integrating these factors to evaluate the hydration process and strength development of concrete [31]. The maturity index is calculated considering the curing time at specific temperatures, making it a crucial variable in predicting strength development.

**Figure 7:**
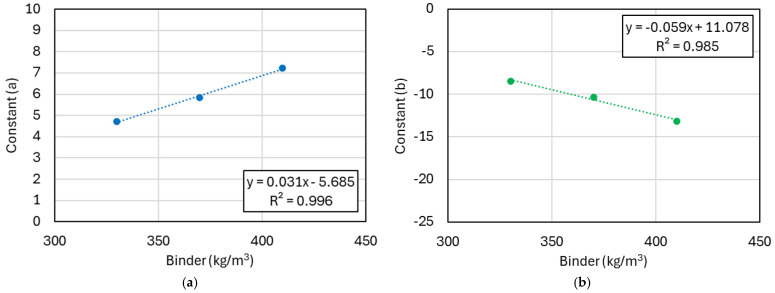

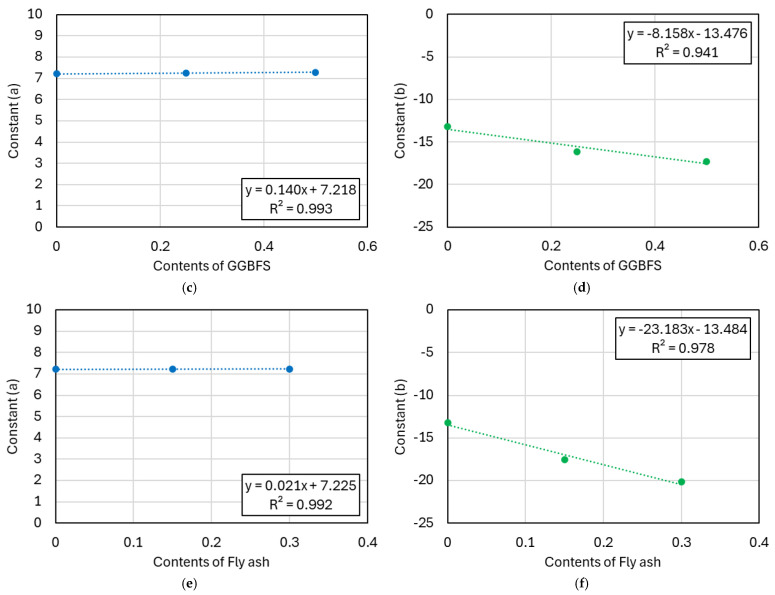
Relationship between concrete mix proportions and strength coefficient. (**a**) a-binder contents; (**b**) b-binder contents; (**c**) a-contents of GGBFS; (**d**) b-contents of GGBFS; (**e**) a-contents of FA; and (**f**) b-contents of FA.

Applying the maturity concept and logarithmic function model allows for effective prediction of concrete strength under varying temperature conditions. By reflecting the changes in constants a and b according to the binder type and content, more accurate strength predictions can be achieved. Figure 7 shows the analysis of the strength coefficients a and b in the logarithmic strength prediction model based on binder content and type. This analysis builds on the regression model results presented in Figure 5 and Figure 6. In the logarithmic model, the coefficient a affects the final compressive strength, while b influences the rate of strength development, determining the slope of the curve [32]. The coefficient a indicates how quickly the compressive strength increases as maturity progresses, representing the ultimate strength the concrete can achieve. On the other hand, b is related to the early strength gain rate, with higher b values indicating faster initial strength development, while lower values suggest a more gradual increase in strength.

The results indicate that as the binder content increases, the coefficient a tends to increase, whereas b decreases. This suggests that a higher binder content leads to greater ultimate compressive strength, reflected in the increase in a, while the strength development rate slows down, causing b to decrease. This can be explained by the characteristic of the logarithmic model, where the increased binder content requires more time for complete hydration, resulting in a higher final strength but a more gradual strength curve.

In mixtures incorporating GGBFS or FA, there is minimal change in a, indicating that the ultimate strength remains relatively stable. However, b decreases as the content of these mineral admixtures increases. This is due to the delayed strength development associated with secondary reactions (e.g., pozzolanic reactions) that occur with these materials. The secondary reactions rely on calcium hydroxide (Ca(OH)_2_) produced during cement hydration, but in the early curing stages, limited Ca(OH)_2_ availability slows down these reactions, resulting in delayed strength gain and lower b values [33].

### 3.3. Error Analysis for Reliability Assessment

After deriving a strength prediction equation based on ASTM C 1074 [34], the reliability of the derived equation was evaluated using Average Absolute Error (AAE), Error of Estimate (EoE), and Average Absolute Error of Estimate (Abs. EoE). These error metrics quantitatively analyze the difference between the predicted strength values and the actual measured values, serving as indicators of the accuracy of the prediction equation [3,35]. It was confirmed that smaller AAE and Abs. EoE values indicate that the derived prediction equation can estimate the actual strength with high reliability. This error analysis effectively verifies the reliability of the derived coefficients.

First, the AAE represents the average of the absolute differences between the actual compressive strength and the predicted strength at a specific maturity. A smaller AAE indicates higher accuracy of the prediction equation.

(3) $$AAE=\frac{\sum_{i}^{n}\left|Est.Strength_{i}-Strength_{i}\right|}{n}$$

The AAE represents the mean of the absolute differences between the predicted and measured compressive strengths for nnn estimations, expressed in MPa. Specifically, $Strength_{i}$ refers to the measured compressive strength for the *i*th maturity index $M_{i}$ (MPa), while $Est.Strength_{i}$ denotes the estimated strength at the same maturity $M_{i}$, obtained from the hyperbolic strength–maturity function (MPa). The variable *n* represents the total number of strength estimations.

(4) $$EoE=\frac{Est.Strength_{i}-Strength_{i}}{Strength_{i}}\times 100$$

(5) $$Abs.EoE=\frac{\sum_{i}^{n}\left|EoE_{i}\right|}{n}$$

Error of Estimate (EoE) indicates whether the predicted strength is overestimated or underestimated compared to the actual strength as a percentage. The sign of the EoE reflects the direction of the error; a positive value indicates overestimation, while a negative value indicates underestimation. To evaluate the magnitude of the error regardless of its direction, the Abs. EoE is used. Abs. EoE averages the absolute values of the EoE, providing an assessment of the overall error magnitude and thereby contributing to the evaluation of reliability, where the EoE for the *i*th test strength is calculated as a percentage (%), and *n* denotes the total number of EoE values considered in the analysis.

**Figure 8:**
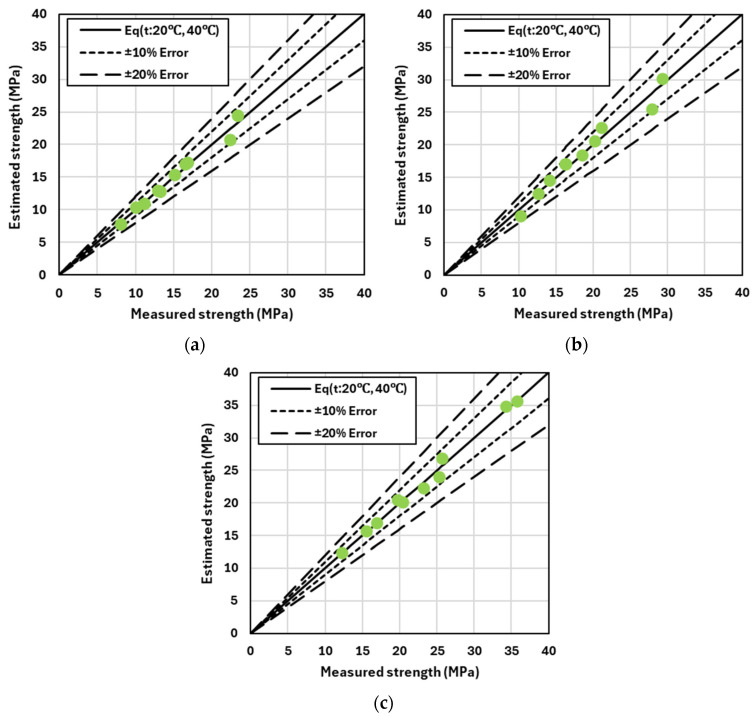
Strength analysis by binder content: measured vs. estimated with ±10% and ±20% error. (**a**) B33; (**b**) B37; and (**c**) B41.

**Figure 9:**
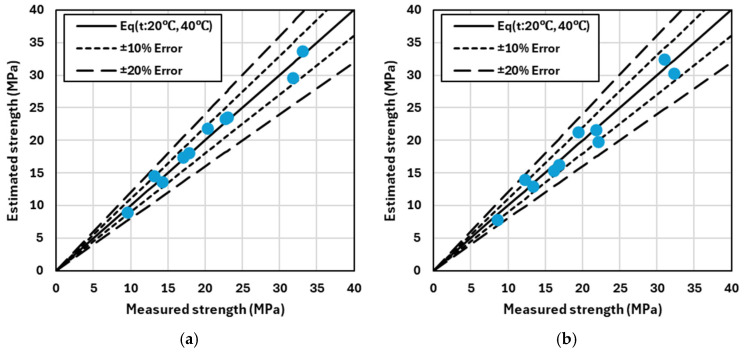

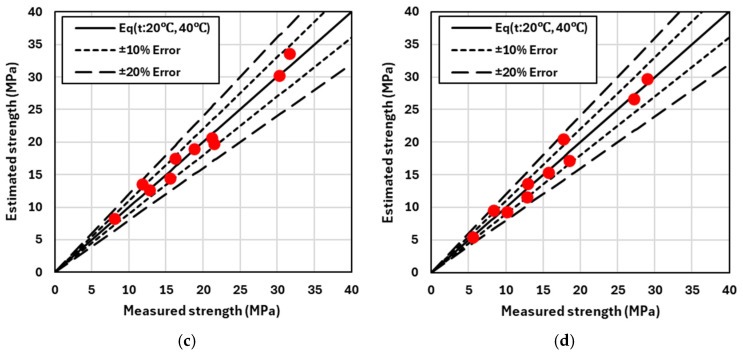
Strength analysis by binder type: measured vs. estimated with ±10% and ±20% error. (**a**) B41_S25; (**b**) B41_S50; (**c**) B41_F15; and (**d**) B41_F30.

Figure 8 and Figure 9 compare the predicted compressive strength with the actual measured compressive strength under curing conditions at 20 °C and 40 °C, respectively. The solid black line in each graph represents the line of equality, where the predicted and actual strengths match. If the predicted strength exceeds the actual strength, the data points lie above this line; conversely, if the predicted strength is lower than the actual strength, the data points fall below the line. The short and long dashed lines indicate error margins of ±10% and ±20%, respectively.

These graphs visually demonstrate how the error between predicted and actual strength varies, particularly as the strength increases. The results reveal that discrepancies between predicted and actual strengths are more frequent in the high-strength range. First, Figure 8 illustrates the difference between the predicted and actual strength as a function of the binder content. When only OPC is used, the Error of Estimate (EoE) generally does not exceed ±10%, regardless of changes in the binder content. This indicates that variations in the binder content have a minimal impact on the error between predicted and actual strength. In contrast, Figure 9 shows the difference between the predicted and actual strength based on the binder type, specifically, mixtures incorporating GGBFS and FA. In this case, data points frequently exceed the ±10% EoE threshold when these supplementary cementitious materials (SCMs) are used, suggesting that the error between the predicted and actual strength is larger when GGBFS or FA is included in the mixture.

Another key observation from the graphs is the trend of increasing error in the high-strength region. Particularly for high-strength concrete, the accuracy of the prediction model decreases, which could be attributed to the characteristics and properties of the supplementary cementitious materials (SCMs) used. These results highlight the importance of considering the binder type and its effects when estimating the predicted strength. Specifically, when using SCMs such as GGBFS or FA, existing prediction models may be less accurate, and additional calibration may be necessary to improve the prediction accuracy.

Table 5 provides a comprehensive summary of the AAE and Absolute Error of Estimate (Abs. EoE) for the compressive strength of concrete mixes, including B33, B37, B41, and various B41-based mixes containing slag and fly ash. This table consolidates the results derived from Figure 8 and Figure 9, comparing the prediction accuracy according to different binder contents and types. As the binder content increases from B33 (330 kg/m^3^) to B41 (410 kg/m^3^), a significant decrease in both AAE and Abs. EoE values is observed. For instance, the AAE decreases from 1.19 MPa for B33 to 0.51 MPa for B41, while the Abs. EoE drops from 8.78% to 2.16%. This trend clearly indicates that as the binder content increases, the alignment between predicted and actual strengths improves, thereby enhancing the reliability of the prediction model.

When slag and fly ash are incorporated into the B41 mix, there is considerable variation in AAE and Abs. EoE values. Specifically, the use of 25% and 50% GGBFS (B41_S25 and B41_S50) results in an increase in AAE values to 1.27 MPa and 1.89 MPa, respectively, and an increase in Abs. EoE values to 7.01% and 12.46%. This suggests that slag, particularly at higher replacement levels, introduces more variability and potential errors in strength predictions, likely due to the different hydration characteristics of slag compared to OPC. Similarly, when FA is partially substituted (B41_F15 and B41_F30), the AAE and Abs. EoE values also increase compared to the B41 mix without SCMs. For B41_F15, the AAE is 0.97 MPa with an Abs. EoE of 5.34%, and for B41_F30, the AAE is 1.06 MPa with an Abs. EoE of 7.24%. Although these values are lower than those observed with slag, they still indicate that the addition of FA impacts the accuracy of the strength prediction model to some extent.

### 3.4. Evaluation of Maturity and Strength for Slab and Beam Concrete Structures

A methodology for predicting strength development as a function of time and temperature was proposed by applying the maturity concept, with a particular focus on estimating the real-time strength based on the heat generated by the hydration reaction of concrete. The maturity was calculated by integrating the cumulative effects of temperature and time under different binder conditions, forming the basis for deriving the strength prediction model in Equation (2). To verify this strength prediction model, mock-up specimens that simulate actual structures were created, with continuous monitoring of the internal temperature to calculate the maturity index.

**Figure 10:**
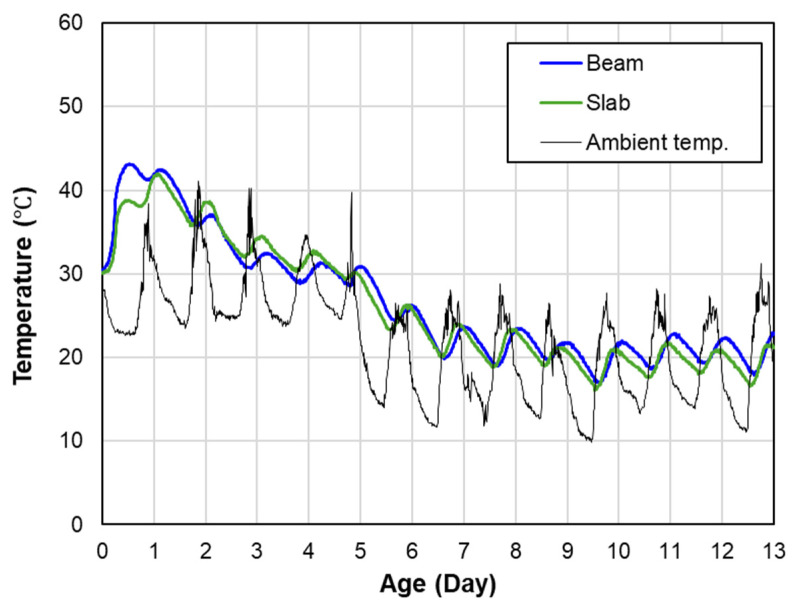
Core temperature of slab and beam structures.

Slab- and beam-shaped structures were created, and temperature data were collected over 13 days using thermometers installed at the center of each structure. Figure 10 illustrates the core temperature variations of slab and beam structures over time, highlighting the influence of hydration heat and ambient temperature fluctuations. Due to the thin cross-sections, the temperature variations in the slab (green line) and beam (blue line) closely followed the periodic fluctuations of the surrounding temperature (black line), showing similar patterns. The initial temperature ranged between 35 °C and 40 °C, and the heat of hydration caused the beam’s temperature to be higher than that of the slab. However, after 24 h, the ambient temperature began to have a greater influence on the concrete.

The maturity values derived from the temperature data for both the slab and beam were similar, resulting in minimal differences between the predicted and actual core strengths. According to Table 6, the analysis of AAE and EoE indicates that the prediction model demonstrates high reliability for both structures. The beam, in particular, exhibited slightly lower errors than the slab, which can be attributed to the beam’s thickness providing some insulation against ambient temperature fluctuations. Additionally, the EoE values remained below 10%, confirming a strong agreement between the predicted and actual strengths. These results indicate that the prediction model shows high reliability and agreement with the actual strengths for both slab and beam structures. The study confirms that the maturity model can provide accurate strength predictions, taking into account the structure’s shape and external conditions. This analysis demonstrates that the prediction model can deliver meaningful results for practical field applications.

## 4. Conclusions

This study presents a comprehensive approach to address the challenges in predicting the compressive strength of concrete under various curing conditions and binder compositions. By using OPC mixed with GGBFS and FA, the study analyzed the relationship between the curing temperature, binder content, and strength development. This research combines the maturity method with a logarithmic function model to predict concrete strength under different temperature conditions, allowing for a more accurate reflection of actual curing environments. As a result, the proposed prediction model showed high coefficients of determination and error rates below 10% at curing temperatures of 20 °C and 40 °C, demonstrating a strong performance.

However, when GGBFS and FA were used as binders, the delayed early strength development led to significant prediction errors under low-temperature conditions. Particularly at curing temperatures of 5 °C, the actual strengths were lower than the predicted ones, indicating difficulties in early strength prediction. This discrepancy is due to the slower reaction of supplementary cementitious materials, which are sensitive to temperature variations during hydration. Therefore, additional calibration may be needed for predicting early strength in mixtures containing GGBFS and FA under low-temperature conditions.

The proposed prediction model was further validated through a mock-up test simulating real structural members such as a slab and beam. The comparison between predicted compressive strengths and measured ones showed minimal differences, confirming the reliability and practicality of the model. This approach overcomes the limitations of previous models and provides a robust framework for accurate strength prediction across various environmental and mix conditions.

In conclusion, this study presents a refined strength prediction model that considers actual field variables often overlooked in laboratory tests, contributing significantly to structural design and construction management. By enabling reliable predictions of concrete performance under diverse field conditions, the proposed model enhances the safety and durability of concrete structures. In future research, the integration of real-time monitoring technologies and imaging techniques will be explored to collect hydration heat data, which will serve as a basis for continuously improving and applying the prediction model in more dynamic curing environments.

## Figures and Tables

**Table 1:** Physical properties and chemical composition of materials used.

Type	Density (g/cm^3^)	Blaine (m^2^/kg)	Chemical Composition								
			CaO	SiO_2_	Al_2_O_3_	Fe_2_O_3_	SO_3_	MgO	K_2_O	Na_2_O	LOI
Cement	3.152	331	69.6	12.8	3.5	5.2	2.3	3.3	2.0	0.5	1.2
GGBFS	2.908	468	43.4	34.6	14.3	0.6	5.0	5.1	0.5	0.2	0.0
FA	2.203	322	3.5	56.8	22.8	6.9	0.5	1.8	1.1	0.8	2.4

**Table 2:** Concrete mix proportions.

Mixture	W/B (%)	S/a (%)	Unit Weight (kg/m^3^)						Super- Plasticizer (kg/m^3^)
			Water	Binder			Fine Aggregate	Coarse Aggregate	
				Cement	GGBFS	FA			
B33	50.0	50.0	165	330	–	–	891	901	0.19
B37	46.5	49.5	169	370	–	–	856	884	0.48
B41	42.0	49.0	172	410	–	–	840	867	0.62
B41_S25	42.0	49.0	172	308	103	–	823	866	0.65
B41_S50	42.0	49.0	172	205	205	–	814	857	0.70
B41_F15	42.0	49.0	172	349	–	62	821	864	0.60
B41_F30	42.0	49.0	172	287	–	123	810	853	0.60

**Table 3:** Concrete maturity and compressive strength at different temperatures by binder content.

Temp.	B33		B37		B41	
	$M_{\left(t,T\right)}$ (Degree∙Day)	Strength (MPa)	$M_{\left(t,T\right)}$ (Degree∙Day)	Strength (MPa)	$M_{\left(t,T\right)}$ (Degree∙Day)	Strength (MPa)
5 °C	49.4	5.9	50.1	7.4	51.4	8.1
	112.1	10.1	112.8	14.1	111.4	16.3
	157.2	12.0	156.9	17.5	157.4	19.3
	218.2	13.8	218.6	20.1	218.3	23.0
	769.9	23.3	766.1	28.9	785	36.0
20 °C	33.6	7.8	33.9	9.1	34.1	12.4
	64.8	10.9	65.4	14.5	65.0	16.9
	94.7	12.9	95.6	17.1	95.9	20.5
	217.4	17.3	219.1	22.6	226.3	26.9
	873.9	24.5	876.9	30.2	882.8	35.6
40 °C	50.9	10.4	50.8	12.5	53.6	15.7
	101.3	12.8	94.3	16.0	104.5	20.2
	151.8	15.4	139.4	18.4	155.2	22.3
	202.2	17.0	185.2	20.6	205.7	24.0
	707.8	20.7	688.4	25.5	713.3	34.8

**Table 4:** Concrete maturity and compressive strength at different temperatures by binder type.

Temp.	B41_S25		B41_S50		B41_F15		B41_F30	
	$M_{\left(t,T\right)}$ (Degree∙Day)	Strength (MPa)	$M_{\left(t,T\right)}$ (Degree∙Day)	Strength. (MPa)	$M_{\left(t,T\right)}$ (Degree∙Day)	Strength (MPa)	$M_{\left(t,T\right)}$ (Degree∙Day)	Strength (MPa)
5 °C	49.5	6.9	49.7	5.5	49.4	3.8	49.3	3.6
	107.5	10.6	107.7	7.8	107.2	9.4	107.0	8.0
	152.0	13.2	152.4	12.3	151.7	11.8	151.4	10.6
	211.0	17.4	211.5	16.3	210.5	16.0	210.1	13.5
	742.4	32.4	744.2	29.2	740.9	24.6	739.4	23.0
20 °C	34.3	7.0	34.6	7.8	34.5	8.3	34.8	5.4
	66.1	11.6	66.7	13.0	65.9	12.7	66.3	9.3
	96.6	15.4	97.5	15.3	97.2	14.4	97.8	11.6
	221.8	23.6	223.5	19.8	229.8	19.7	230.9	17.2
	891.4	33.7	894.4	30.3	898.8	33.6	900.5	29.7
40 °C	57.0	16.6	53.4	14.0	57.4	13.5	51.8	9.5
	113.5	20.1	104.3	16.3	111.9	17.5	96.2	13.6
	170.0	23.9	155.1	21.3	166.1	19.0	142.2	15.4
	226.4	23.3	205.6	21.6	220.0	20.6	188.9	20.5
	792.7	29.6	713.7	32.5	763.2	30.2	702.2	26.6

**Table 5:** AAE and Abs. EoE analysis results by concrete compressive strength mix conditions.

Type	B33	B37	B41	B41_S25	B41_S50	B41_F15	B41_F30
AAE (MPa)	1.19	0.80	0.51	1.27	1.89	0.97	1.06
Abs. EoE (%)	8.78	4.39	2.16	7.01	12.46	5.34	7.24

**Table 6:** Strength prediction and error analysis for slab and beam structures.

Type	Age (Day)	Maturity (MM)	Predicted Strength	Core Strength (MPa)	AAE (MPa)	EoE (%)
Slab	13	340.9	28.9	26.4	2.52	9.56
Beam	13	347.3	29.1	26.9	2.16	8.03

## Data Availability

The original contributions presented in the study are included in the article, further inquiries can be directed to the corresponding author.
